# A Case Report of Leptomeningeal Myelomatosis and Rapid Improvement with Regimen Consisting of Daratumumab, Pomalidomide, Vincristine, Procarbazine, and Dexamethasone

**DOI:** 10.1155/2022/4081971

**Published:** 2022-08-31

**Authors:** Jew Win Kuan, Sing Ling Chai, Pathmanathan Rajadurai, Lee Gong Lau, Joseph Uchang, Sharifah Noor Akmal Syed Husain

**Affiliations:** ^1^Department of Medicine, Universiti Malaysia Sarawak, Kota Samarahan, Sarawak, Malaysia; ^2^Timberland Medical Centre, Kuching, Sarawak, Malaysia; ^3^Subang Jaya Medical Centre, Subang Jaya, Selangor, Malaysia; ^4^Borneo Medical Centre, Kuching, Sarawak, Malaysia; ^5^Pantai Premier Pathology Sdn Bhd, Kuala Lumpur, Malaysia

## Abstract

Central nervous system (CNS) involvement in multiple myeloma (MM) (MM-CNS) in the form of leptomeningeal myelomatosis or brain parenchyma plasmacytoma is rare, causing challenges in clinical diagnosis and treatment. We would like to report a case of leptomeningeal myelomatosis and illustrated the challeges. A 61-year-old man was diagnosed with MM with left paravertebral plasmacytoma, R-ISS II with high suspicion of double-hit MM, either biallelic aberrancy of TP53 or del(17p) and IGH aberrancy depending on the definition chosen, treated with lenalidomide-bortezomib-dexamethasone and local radiotherapy, later developed systemic relapse and progression to MM-CNS in the form of leptomeningeal myelomatosis. A modified CNS-based treatment not reported before, consisting of daratumumab, pomalidomide, vincristine, procarbazine, and dexamethasone, brought a rapid clinical improvement and warrants a further study. Incorporation of intrathecal thiotepa into the regimen would likely increase the efficacy.

## 1. Introduction

Central nervous system (CNS) is much less involved in multiple myeloma (MM) (MM-CNS) compared to other haematological neoplasms like acute leukaemia and lymphoma. Cranium osseous or osteodural plasmacytoma that usually results from the spread of contiguous bone lesion should be separated from MM-CNS because it is common and has a better prognosis than MM-CNS (median overall survival (OS): 25 months (*n* = 38) versus 6 months (*n* = 12)) [1].

MM-CNS could be in the form of leptomeningeal myelomatosis (usually with cerebral spinal fluid (CSF) plasmatocytosis) or, rarer, brain parenchyma plasmacytoma (usually without CSF plasmacytosis). It can present at diagnosis or relapse/progression of MM, rarer in the former—16% at diagnosis [2]. This rarity could be an underestimation because CNS assessment is not a routine MM diagnostic workout unless there was CNS symptom, which if present, might also be overlooked because the treating physician might attribute the CNS symptom to the localised bony lesion, hypercalcaemia, uraemia, paraproteinaemia, concomitant disease e.g. psychological disorder, and adverse effects of MM or non-MM drugs.

## 2. Case Presentation

**Figure 1:**
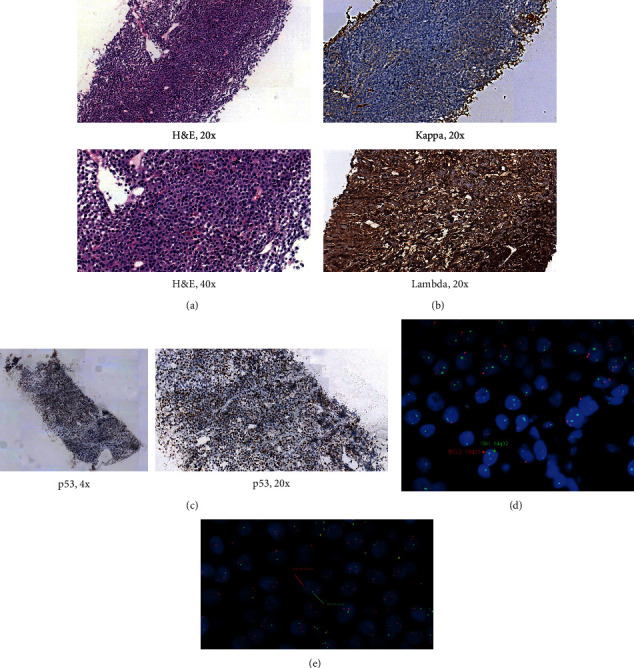
Left paravertebral plasmacytoma biopsy on (a) haematoxylin and eosin (H&E) stain, (b) immunohistochemical (IHC) stain of kappa and lambda light chain, (c) IHC stain of p53, (d) FISH of IGH breakapart, and (e) FISH of TP53 deletion.

A 61-year-old man with underlying hypertension and anxiety disorder presented with backache and left lower chest pain since end of year 2020 and later was diagnosed with MM with left T8-T11 paravertebral plasmacytoma in March 2021. There was no bone marrow examination because patient refused. CT-guided biopsy of left lower thoracic paravertebral mass showed plasmacytoma with lambda light chain restriction (Figure 1). MRI thoracic spine showed left T8-T11 posterior mediastinal soft tissue mass, with a size of 6.8 × 3.8 × 10.0 cm, with intraspinal extension and infiltration into left posteromedial segment of 10^th^ and 11^th^ rib (Supplementary Data (available here)). Serum protein electrophoresis (SPE) and immunofixation (IF) showed IgA lambda of 10 g/L without immunoparesis, while urine protein electrophoresis (UPE) and IF did not show evidence of paraprotein. Serum free light chain (sFLC) showed reduced kappa:lambda ratio of 0.10 (13.2 mg/L:132.7 mg/dL). Other investigations were normal—beta2-microglobulin 1.7 mg/L, albumin 42 g/L, Hb 15.7 g/dL, TWC 4.547 × 10^9^/L, PLT 219.9 × 10^9^/L, creatinine 83 *µ*mol/L, eGFR 87 mL/min/1.73 m^2^, corrected Ca 2.37 mmol/L, and LDH 172 *µ*/L within normal range.

Patient developed major depressive disorder since the diagnosis of MM on top of the preexisting anxiety disorder. Despite psychological therapy, both conditions waxed and waned following the condition of MM. Hence, throughout the follow-up, it is difficult to manage this patient, especially on the evaluation of certain subjective symptoms.

Dexamethasone was started at the end of March 2021 while waiting for the results of the investigations above. Bortezomib-dexamethasone plus zoledronic acid (VD + *Z*) was started in the middle of April 2021 instead of the usual triplet with addition of cyclophosphamide, thalidomide, or lenalidomide (*R*) due to patient's concern on potential adverse effect of all drugs and to keep the number of drug minimum with intention to increase to triplet once proven he was tolerable to VD + *Z*. In early May 2021, MRI thoracic spine showed progression of the paravertebral plasmacytoma, with a size of 8.2 × 5.8 × 15 cm, without significant change in the involvement at T9/10 and T10/11 foramina and intraspinal extension (Supplementary Data). In the middle of May 2021, local radiotherapy to the left paraspinal plasmacytoma, 30 Gy in10 fractions, was completed, while systemic MM treatment was changed to RVD + *Z*. Retrospective investigation of the initial paravertebral plasmacytoma sample revealed that about 30% of the tumour cells showed aberrant nuclear p53 protein expression (Figure 1), positivity for fluorescent in situ hybridisation (FISH) TP53 deletion (nuc ish (DLEU × 2,TP53 × 1)(200)) (Figure 1), and positivity for FISH IGH rearrangement (nuc ish (IGH × 3,BCL × 2)(51/200)) (Figure 1).

After 2^nd^ cycle of RVD + *Z* at the end of June 2021, patient achieved at least a partial response (PR) [3]. SPE showed IgA lambda of <1 g/L. UPE showed a faint band at fast moving gamma region. sFLC showed normal ratio of 1.04 (18.8 mg/L:18.0 mg/L). MRI and PETCT showed reduction of the paravertebral mass (Supplementary Data).

After 4^th^ cycle of RVD + *Z* in the middle of August 2021, patient achieved a very good PR (VGPR) or near complete response (CR) [3]. SPE and IF were negative, while UPE showed a faint restriction band at fast moving gamma region for which IF did not reveal any abnormality. sFLC showed normal ratio of 1.22 (15.4 mg/L:12.6 mg/L). MRI and PETCT showed further reduction of the paravertebral mass (Supplementary Data).

The patient was a heavy smoker. During treatment of MM, imaging showed occurrence of lung changes which were progressive (Supplementary Data). He was investigated and treated as smear-negative pulmonary tuberculosis (PTB) due to clinical suspicion and endemicity of PTB in the region. Anti-TB was started in August 2021, while MM treatment was changed to lenalidomide maintenance. The lung changes improved with anti-TB (Supplementary Data).

In November 2021, SPE/IF, UPE/IF, and sFLC remained negative and PETCT showed stable left paraspinal mass. His anti-TB was continued until the diagnosis of leptomeningeal myelomatosis in February 2022.

**Figure 2:**
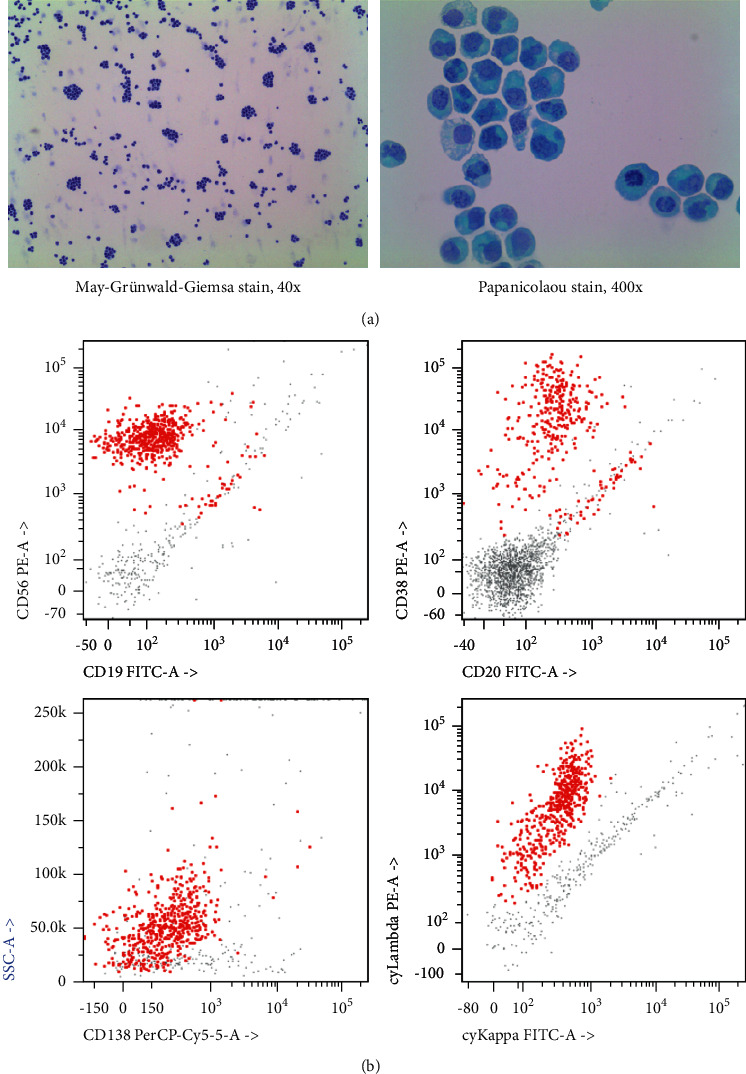
Cerebrospinal fluid examination at diagnosis (February 2022): (a) cytology and (b) immunophenotyping (the neoplastic plasma cells CD38+/CD56+/CD138-/lambda-restricted were gated red).

His backache and peripheral sensory neuropathy worsened around December 2021. He was diagnosed with sciatica due to nerve root compression and received a steroid injection to left L4/4 facet joint at the end of December 2021, but the injection did not improve his condition. Lenalidomide maintenance was off in the middle of January because worsening drug-induced peripheral neuropathy was suspected while anti-TB, which could also cause drug-induced peripheral neuropathy, was continued to ascertain the main causative agent and in view of the importance of complete anti-TB regimen. However, symptoms were not improved and motor weakness at right wrist and fingers appeared in February 2022. MRI brain and whole spine in the middle of February 2022 showed multiple nodular lesions at leptomeningeal at the cerebrum, cerebellum, spinal cord, cauda equina, and bilateral internal auditory canals (Supplementary Data). CSF analysis showed protein 1.83 g/L, glucose 3.3 mmol/L, cell count 113 × 10^6^/L with all mononuclear cells, cytology of abundant large atypical plasmacytoid cells in loose clusters along with singly dispersed cells, and immunophenotyping of lambda-restricted plasma cells (Figure2). SPE showed reappearance of M-protein <1 g/L. He was treated as MM-CNS at relapse/progression in this case given the tempo of the worsening CNS symptoms which appeared more than a year after initial diagnosis of MM, although MM-CNS at diagnosis could not be confidently ruled out because no CNS assessment was done.

**Figure 3:**
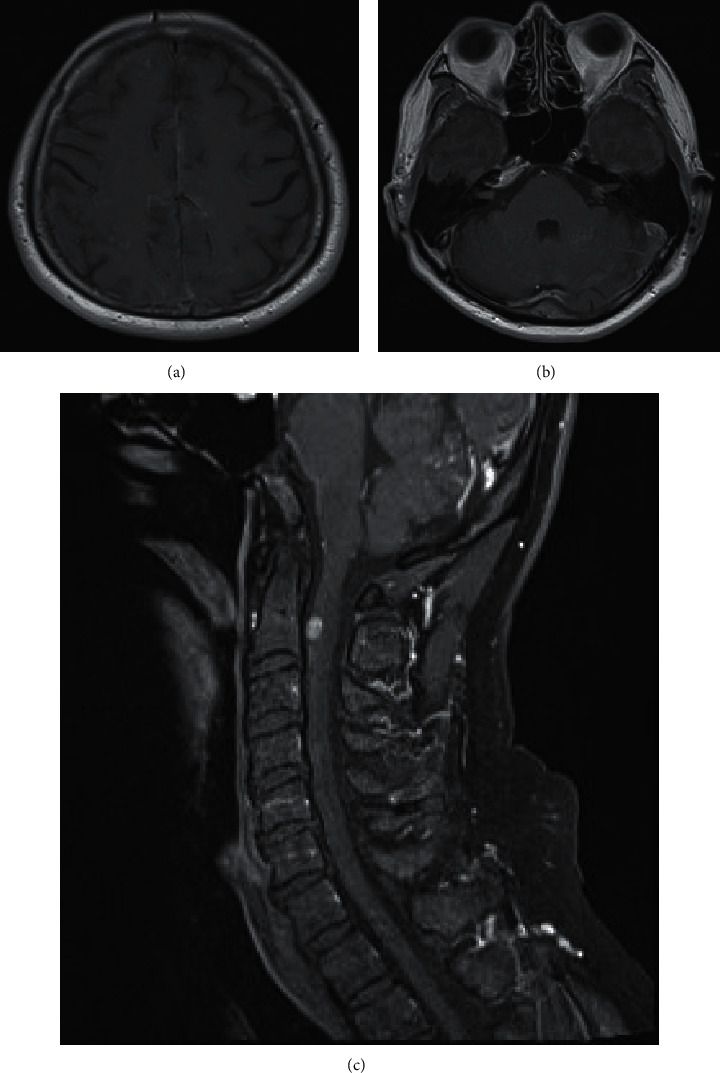
MRI of leptomeningeal myelomatosis at progression of leptomeningeal myelomatosis (March 2022): (a) T1 with contrast, vertex level, (b) T1 with contrast, level at bilateral IAC lesions, and (c) T1 with contrast, cerebellum and cervical spine.

After the diagnosis of leptomeningeal myelomatosis, he received intravenous methylprednisolone 1 g daily for three days which improved the symptoms partially. He decided to take traditional treatment, which did not improve the symptoms further instead the symptoms started to worsen after a week. At the end of March 2022, he and his family members agreed for chemotherapy. Before the start of chemotherapy, he was wheelchair and bed bound with lower limbs' power of grade 3 at the right and 4 at the left and upper limbs' power of 3-4 at the right and 4-5 at the left. Finger-nose test was positive. He was delirious and had visual and auditory hallucination. MRI showed worsening of leptomeningeal lesions (Figure 3). Dara-PVPD (daratumumab, pomalidomide, vincristine, procarbazine, and dexamethasone) was started. His condition was rapidly improved. He was able to stand and walk with walking frame after two cycles of treatment. At the time of writing, he completed the 4^th^ cycle in the middle of May 2022. Between the 2^nd^ and 3^rd^ cycle, he developed an abscess at left gluteal ischial tuberosity region which resolved with antibiotic and local surgical drainage but resulted a week delay to the commencement of the 3^rd^cycle. The peripheral sensory neuropathy worsened from fingertips to whole palms and soles after the 2^nd^ cycle needing dose modification. The detail of Dara-PVPD given from the 1^st^ to 4^th^ cycle is shown in Supplementary Data.

He achieved a VGPR^3^ after the 4^th^ cycle. CSF analysis in the middle of the 4^th^ cycle showed protein 0.58 g/L, glucose 3.2 mmol/L, cell count 6 × 10^6^/L, cytology of low cellularity with no malignant cell seen, and immunophenotyping of no evidence of clonal plasma cells and myeloma involvement. MRI showed disappearance of leptomeningeal lesion in the cerebrum, cerebellum, spinal cord, and cauda equina, except PR at left internal auditory canal and left external capsule (Supplementary Data).

He completed 6^th^ cycle in the middle of June 2022. Assessment post-6^th^ cycle showed further CNS improvement—CR with CSF and other MRI lesions except stable external capsule lesion as compared to MRI post-4^th^ cycle. At the time of writing, he was undergoing peripheral blood haematopoietic stem cell collection and planned for thiotepa-based autologous haematopoietic stem cell transplantation in the middle of July 2022.

## 3. Discussion

This case illustrated the diagnostic difficulty of leptomeningeal myelomamastosis like the other reported cases, but further complicated by the psychological disorders and neurotoxicity effect of anti-TB. The MM drugs which commonly cause CNS adverse effects and were used in this patient are bortezomib, lenalidomide, and dexamethasone. Bortezomib causes peripheral neuropathy 28-41%, neuralgia/pain in limb 8-34%, paresthesia and dysesthesia 9-16%, headache 4-13%, and dizziness 3-5% more than the control group [4]. Lenalidomide causes peripheral neuropathy (3–5%), neuralgia/pain in limb (3–11%), paresthesia and dysesthesia (3–7%), headache (5–20%), dizziness (4–20%), muscle cramp (12–18%), back pain (7–21%), and bone pain (2%) [5]. We are aware of the potential CNS and musculoskeletal adverse effects of Dara-PVPD. However, after diagnosis of leptomeningeal myelomatosis was made, more objective assessments like CSF analysis and imaging would greatly assist in evaluation of symptoms.

Revised International Staging System (R-ISS) is a widely used risk stratification approach in MM which incorporates high risk chromosomal abnormalities (CAs)—del(17p), t(4; 14), or t(14; 16), but not 1 q gain or mutated TP53 as the data were not available [6]. Walker et al. identified a group (6.1%) in newly diagnosed MM with the highest risk, labelled as double-hit which was defined as having either biallelic inactivation of TP53 (at chromosome 17p) or ISS stage III with CKS1B (at chromosome 1 q) amplification [7]. Mayo Clinic proposed double or triple hit MM as having two or three of the CAs—t (4; 14), t (14; 16), t (14; 20), del 17p, p53 mutation, gain 1 q, and del ( 1p), respectively [8]. FISH can detect del(17p), t(4;14), t(14;16), t(14;20), gain 1q, and del(1p) with high sensitivity and specificity in MM if the sample has high number of plasma cell or was CD138+ cells sorted or if the test has simultaneous CD138 immunofluorescence.However, FISH cannot detect TP53 mutation and CKS1B amplification which are detected using next generation sequencing that is not readily available in our setting. Our patient was likely to have biallelic TP53 aberrancy because his paravertebral plasmacytoma clearly had TP53 deletion at one allele and probably TP53 mutation at the other allele on the argument that MM patients with del17p are likely to have biallelic TP53 aberrancy [9,10] and about 30% of the tumour cells showed aberrant nuclear p53 protein expression. The half-life of the mutated p53 protein is longer compared to the wild type p53 protein which facilitates its detection. Presence of nuclear p53 protein, except nonsense mutation, is highly concordant with TP53 mutation in acute myeloid leukaemia, acute lymphoid leukaemia, myelodysplastic syndrome, or chronic lymphocytic leukaemia [11], less in lymphoma [12–14], and probably also in MM [15]. Nonetheless, p53 nuclear expression correlates with hemizygous TP53 deletion [16, 17] and predicts an adverse outcome [15, 17]. Our patient also had IGH (at chromosome 14q) aberrancy although could not ascertain the aberrancy was t(4;14), t(14;16), or t(14;20). Could there be a discrepancy of result if bone marrow sample was available? We would not be able to answer the question but certainly the sample with highest number of neoplastic plasma cells should be chosen, like the paravertebral plasmacytoma in this case when simultaneous CD138 immunofluorescence was not available.

There is no standard treatment for MM-CNS. Given the poor reported median OS after diagnosis of MM-CNS, 3.5 months (*n* = 9) [18], 4.6 months (*n* = 37) [19], 6 months (*n* = 12) [1], and 6.7 months (*n* = 172) [20], the treatment regimen, Dara-PVPD, was built after consideration of myeloma-effective drugs with CNS penetration. Dara-PVPD was based on a 2-weekly immunochemotherapy regimen used in Primary Diffuse Large B-cell Lymphoma of the CNS, R-MPV (rituximab, methotrexate, procarbazine, vincristine, dexamethasone, intrathecal (IT) or intra-Ommya methotrexate) (Supplementary Data) which was evolved from an established chemotherapy regimen, DeAngelis Protocol21 (Supplementary Data) [21] [22, 23].

Daratumumab, which takes the place of rituximab, has been used in combination with pomalidomide and dexamethasone, first in systemic relapsed and/or refractory MM treatment [24]. Both drugs have CNS penetration [25–27], although daratumumab is likely to have a suboptimal penetration given a case report of leptomeningeal myelomatosis which occurred while on daratumumab maintenance [26]. Daratumumab has been used at a shorter interval, weekly in cycle 1 and 2 of some regimens [24, 28]. Methotrexate was omitted because of its low efficacy in myeloma, while vincristine and procarbazine were retained because of their anti-myeloma activity [29]. IT methotrexate was omitted, again, for the same reason. Other IT agents commonly used in lymphoma were cytarabine and dexamethasone. However, cytarabine is ineffective against myeloma although there was a case report which might support its use [26], while dexamethasone has a doubtful effect as a single agent. IT thiotepa is probably a better choice as a case series reported a median OS of 17 months (*n* = 13) [30]. Thus, IT thiotepa was incorporated in Dara-PVPD but not given during 1^st^ to 6^th^ cycle because the patient was not cooperative for the procedure during the first two cycles and was waiting for import permit and arrival of the drug.

In summary, this case underlined the difficulties to identify CNS involvement in MM in clinical practice and treat the patient in the optimal way. The proposed daratumumab-based regimen could represent a new strategy to treat MM-CNS. Large prospective studies are warranted to identify the optimal therapy for MM-CNS.

## Data Availability

The data used to support the findings of this study are available from the corresponding author upon request.

## Supplementary Materials

Supplementary Materials (1) Supplementary Figure 1 (Serial MRI of the Left Paravertebral Mass): page 2. (2) Supplementary Figure 2 (Serial PETCT of the Left Paravertebral Mass): page 4. (3) Supplementary Figure 3 (Serial CXR of the Lung Lesions): page 5. (4) Supplementary Figure 4 (Serial PETCT of the Lung Lesions): page 7. (5) Supplementary Figure 5 (Serial MRI of the Leptomeningeal Myelomatosis): page 10.(6) DeAngelis Protocol: page 17. (7) R-MPV Protocol: page page 17. (8) Dara-PVPD Protocol: page 18.
