# Supplementary material for: A Case Report of Leptomeningeal Myelomatosis and Rapid Improvement with Regimen Consisting of Daratumumab, Pomalidomide, Vincristine, Procarbazine, and Dexamethasone
Source: Case Rep Hematol. 2022 Aug 31;2022:4081971. doi: 10.1155/2022/4081971 (PMC9453015; doi:10.1155/2022/4081971)
Supplement: Supplementary Materials — (1) Supplementary Figure 1 (Serial MRI of the Left Paravertebral Mass): page 2. (2) Supplementary Figure 2 (Serial PETCT of the Left Paravertebral Mass): page 4. (3) Supplementary Figure 3 (Serial CXR of the Lung Lesions): page 5. (4) Supplementary Figure 4 (Serial PETCT of the Lung Lesions): page 7. (5) Supplementary Figure 5 (Serial MRI of the Leptomeningeal Myelomatosis): page 10.(6) DeAngelis Protocol: page 17. (7) R-MPV Protocol: page page 17. (8) Dara-PVPD Protocol: page 18. [file 4081971.f1.docx]

**Supplementary Data**

Contents

[Supplementary Figure 1 (Serial MRI Of The Left Paravertebral Mass) 2](#_Toc109302289)

[Supplementary Figure 2 (Serial PETCT Of The Left Paravertebral Mass) 4](#_Toc109302290)

[Supplementary Figure 3 (Serial CXR Of The Lung Lesions) 5](#_Toc109302291)

[Supplementary Figure 4 (Serial PETCT Of The Lung Lesions) 7](#_Toc109302292)

[Supplementary Figure 5 (Serial MRI Of The Leptomeningeal Myelomatosis) 10](#_Toc109302293)

[DeAngelis Protocol 17](#_Toc109302294)

[R-MPV Protocol 17](#_Toc109302295)

[Dara-PVPD Protocol 18](#_Toc109302296)

# Supplementary Figure 1 (Serial MRI Of The Left Paravertebral Mass)

| (a) | (b) |
| --- | --- |
| 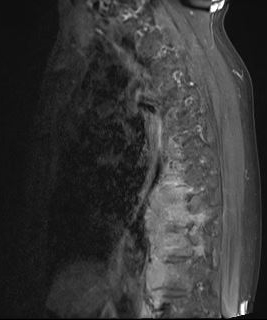  T1 fat saturated image with contrast | 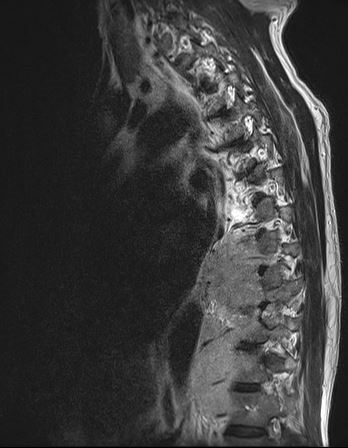  T2 image without contrast |
| (c) | (d) |
| 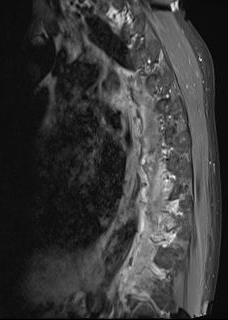  T1 fat saturated image with contrast |   T1 image without contrast |

Supplementary Figure 1. Serial MRI of the left paravertebral mass (a) at diagnosis of multiple myeloma – March 2021, (b) at progression of left paravertebral mass – May 2021, (c) after 2^nd^ cycle of RVD+Z – June 2021, and (d) at diagnosis of leptomeningeal myelomatosis – February 2022

Note:

1. MRI at diagnosis of multiple myeloma – March 2021 showed well defined T8-T11 left paravertebral soft tissue mass, size of 6.8 x 3.8 x 10.0 cm. Anteriorly, it is abutting onto posterior wall of descending thoracic aorta at T9 level with anteriorly displaced thoracic aorta. Medially, there is intraspinal extension via left T9/T10 and T10/T11 neuroforaminae causing mild displacement of the thecal sac to the right. Posteriorly, the mass appears to be limited by the left posterior rib. Left posteromedial segment of the 10^th^ and 11^th^ ribs are likely infiltrated as they have T1W hypointense appearance. There are several oval mildly enhancing lesions with T1W hypointensity and T2 STIR hyperintensity in T7, T8, and T9 vertebral bodies.
2. MRI at progression of left paravertebral mass – May 2021 showed larger left paravertebral soft tissue mass, size of 8.2 x 5.8 x 15 cm, spanning from T8 to T12 levels. At T10 level, there is more than 180 degrees circumferential wall encasement of descending aorta. The aorta is much displaced anteriorly by the mass. The involvement at left T9/T10 and T10/T11 foramina and intraspinal involvement has not changed significantly from previous MRI. The left posteromedial segment of the 10^th^ and 11^th^ ribs lesions are similar to previous MRI. The T8 and T9 vertebral bodies lesions are similar to previous MRI, but T7 lesion is much smaller.
3. MRI after 2^nd^ cycle of RVD+Z – June 2021 showed smaller T8 to T12 left paravertebral soft tissue mass, size of 4.3 x 3.9 x 12.3 cm. The main bulk is only seen at lower T10 level which abuts onto left lateral margin of T10 vertebral body and partially encasing posterior margin of thoracic aorta anteriorly. The intraspinal extension via left T9/T10 and T10/T11 neuroforaminae is much smaller too. There is residual smaller soft tissue lesion along adjacent left 10^th^ rib, size of 5.5 x 0.9 cm. Focal enhancing T1W hypointense lesion in left 10^th^ rib is smaller too. There is no obvious T1W hypointense lesion in left 11^th^ rib now. The T7 vertebral lesion is enhancing and smaller, size of 1.1 cm. T8 vertebral lesion is not enhancing. T9 vertebral lesion shows mild enhancement.
4. MRI at diagnosis of leptomeningeal myelomatosis – February 2022 showed minimal enhancing soft tissue in T9/T10 and T10/T11 neuroforaminae appears similar, compared to previous MRI June 2021. The lateral extension into left T9/T10 and T10/T11 intercostal spaces appear smaller. The enhancing T1W hypointense lesion in left posterior 10^th^ rib is less enhancing now. The T7 enhancing bony lesion is smaller, size 0.7 cm. The T8 and T9 lesions with mild enhancement appear similar.
5. MRI after 4^th^ cycle of Dara-PVPD – May 2022 **(figure not included)** showed the minimal enhancing soft tissue in left T9-T11 paravertebral region appears similar, size of 7.2 x 2.9 x 0.5 cm with axial section measured at mid T11 level. The mild extension into left T9/T10, T10/T11 neuroforaminae and adjacent intercostal spaces appear similar.

# Supplementary Figure 2 (Serial PETCT Of The Left Paravertebral Mass)

| (a) | (b) | (c) |
| --- | --- | --- |
|  |  |  |

Supplementary Figure 2. Serial PETCT of the left paravertebral mass (a) after 2^nd^ cycle of RVD+Z – June 2021, (b) after 4^th^ cycle of RVD+Z – August 2021, and (c) during lenalidomide maintenance – November 2021

Note:

1. PETCT after 2^nd^ cycle of RVD+Z – July 2021 showed residual mild FDG-avid left paraspinal mass (SUVmax of 2.08 and size of 3.1 x 1.8 x 11.7 cm), absence of FDG-avid intraspinal extension, absence of anterior displacement of descending aorta. No abnormal hypermetabolism seen at previously seen local infiltration at posteromedial segment of left 10^th^ and 11^th^ rib. T7 vertebral lesion shows no abnormal FDG avidity. There is a lytic lesion with central sclerosis in right ilium (SUVmax 1.71).
2. PETCT after 4^th^ cycle of RVD+Z – August 2021 showed stable left paraspinal mass but reduced in size (SUVmax of 2.14 and size of 2.5 x 1.7 x 11.7 cm). Reduced avidity at right ilium lesion (SUVmax 1.18).
3. PETCT during lenalidomide maintenance – November 2021 showed stable left paraspinal mass (SUVmax of 2.18 and size of 2.5 x 1.7 x 10.8 cm). Stable right ilium lesion (SUVmax 1.15).

# Supplementary Figure 3 (Serial CXR Of The Lung Lesions)

| (a) | (b) | (c) |
| --- | --- | --- |
| 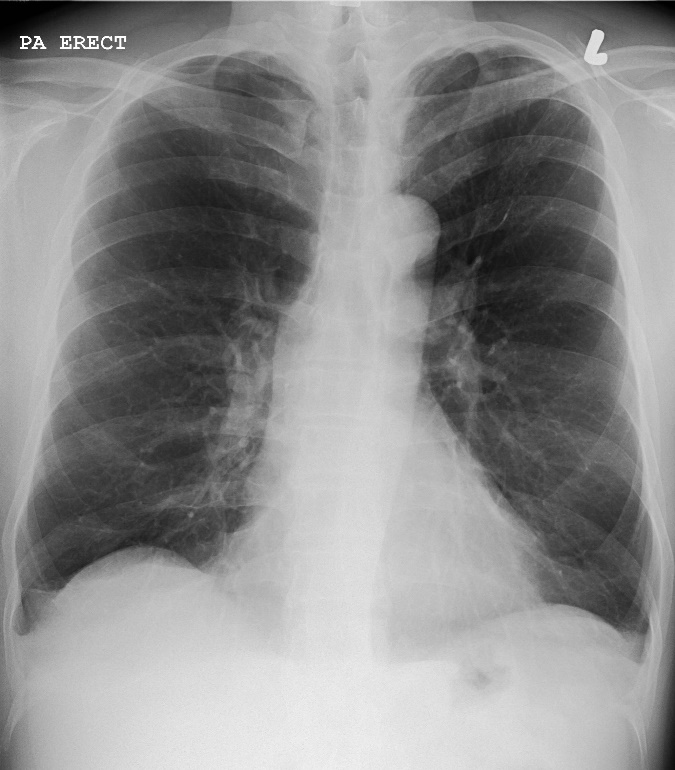 | 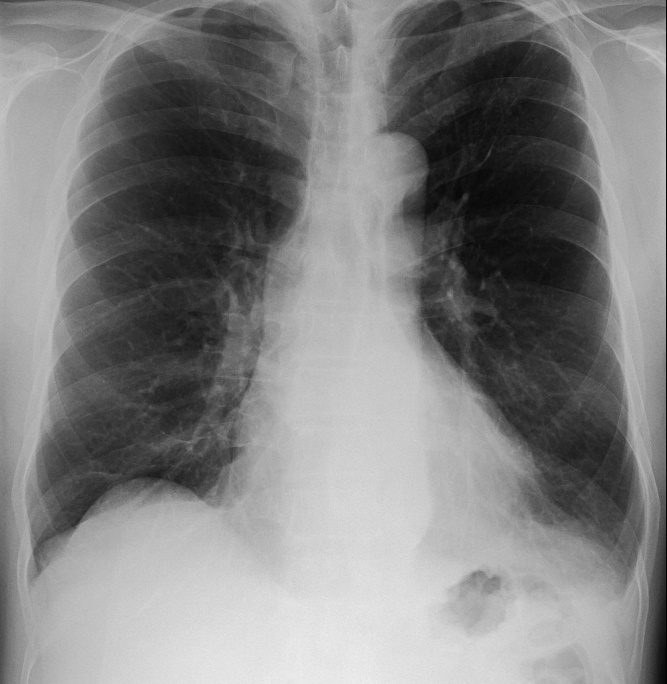 | 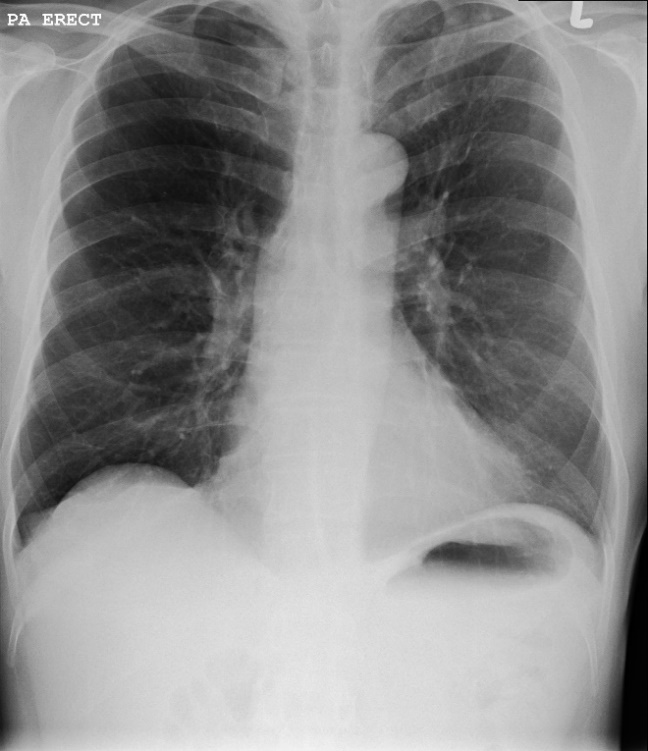 |

| (d) | (e) |
| --- | --- |
| 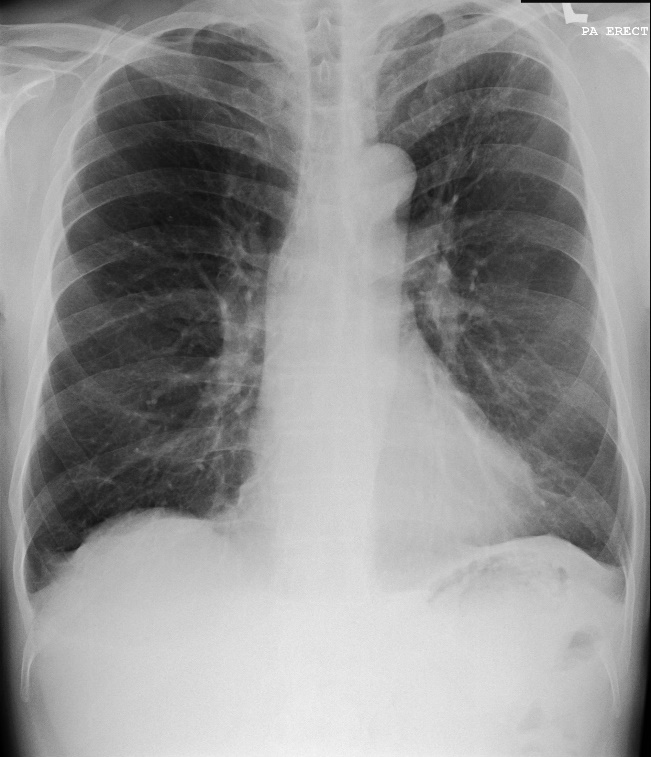 | 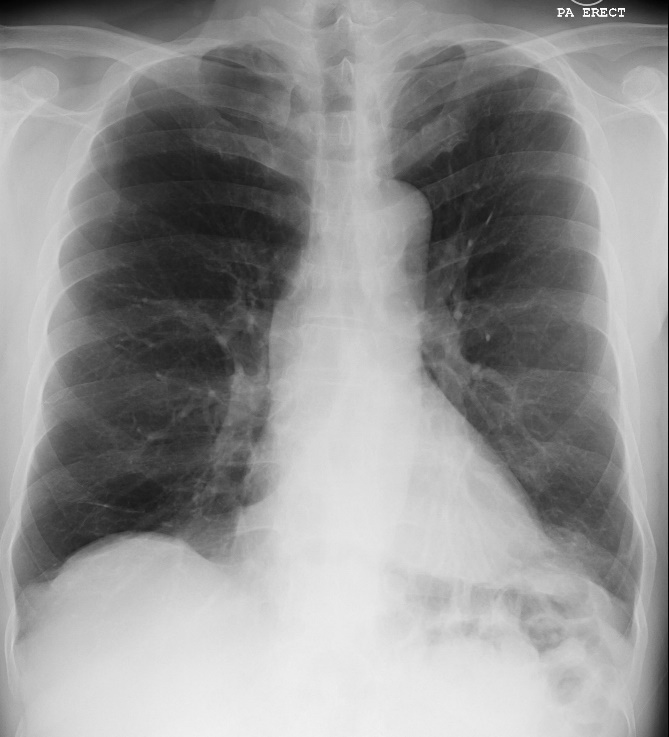 |

Supplementary Figure 3. Serial chest X-ray (a) at baseline – August 2020, (b) at diagnosis of multiple myeloma – March 2021, (c) after radiotherapy of left paravertebral mass – May 2021, (d) after 4^th^ cycle of RVD+Z – August 2021, and (e) during leptomeningeal diagnosis – February 2022

Note:

1. CXR at baseline – August 2020 showed hyperexpanded lung field with bilateral mild reticular opacities.
2. CXR at diagnosis of multiple myeloma – March 2021 showed similar findings as CXR at baseline – August 2020.
3. CXR after radiotherapy of left paravertebral mass – May 2021 showed slightly increased left apical patchy opacities.
4. CXY after 4^th^ cycle of RVD+Z – August 2021 showed further increased left apical patchy opacities.

CXR during anti-TB maintenance – January 2022 **(figure not included)** showed reduced left upper zone reticular fibrotic streaks.

1. CXR during leptomeningeal diagnosis – February 2022 showed reduced left upper zone reticular fibrotic streaks.

# Supplementary Figure 4 (Serial PETCT Of The Lung Lesions)

| (a) | (b) | (c) |
| --- | --- | --- |
| 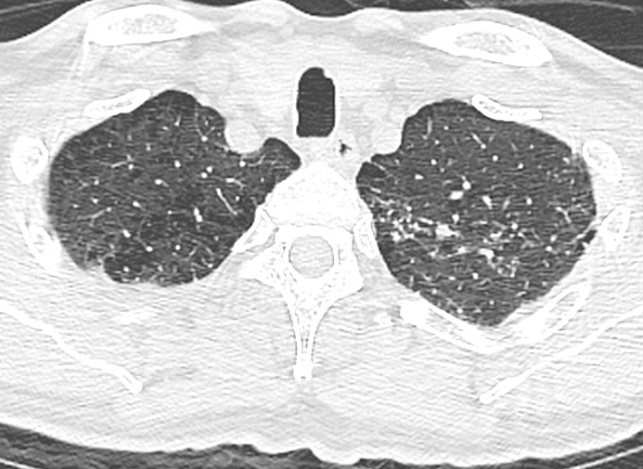  Left upper lobe nodules  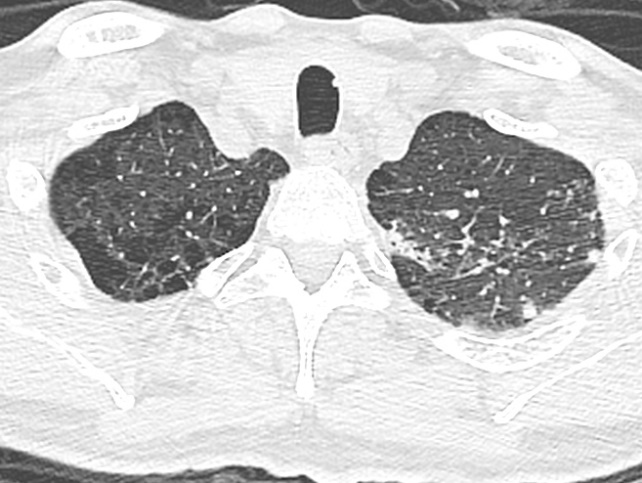  Left upper lobe nodules 2  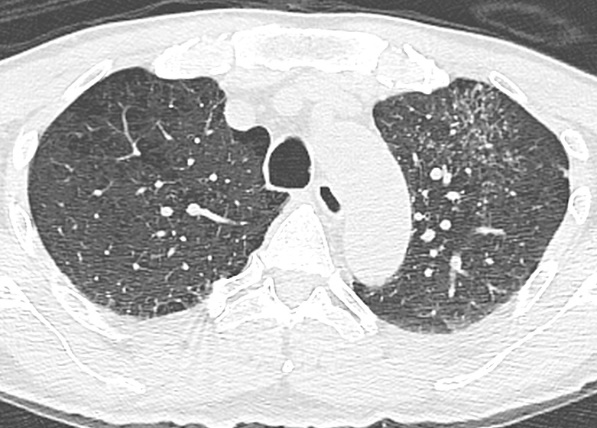  Reticular opacity  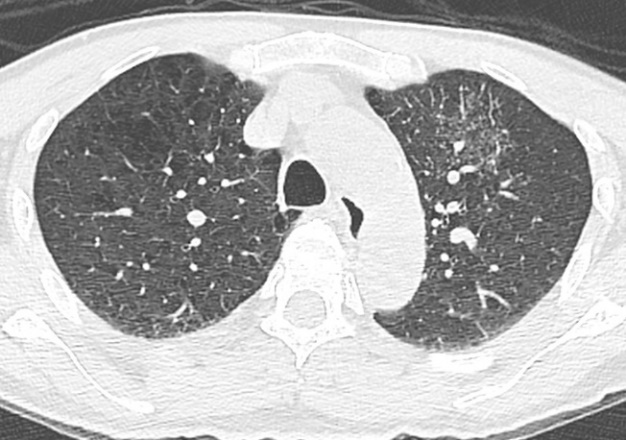  Reticular opacity 2 | 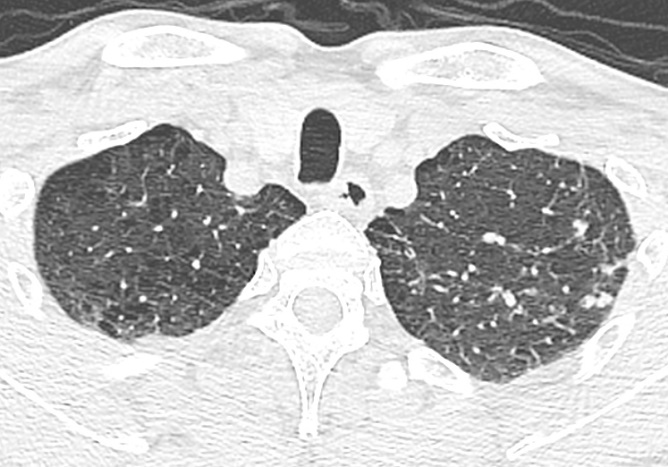  Left upper lobe nodules  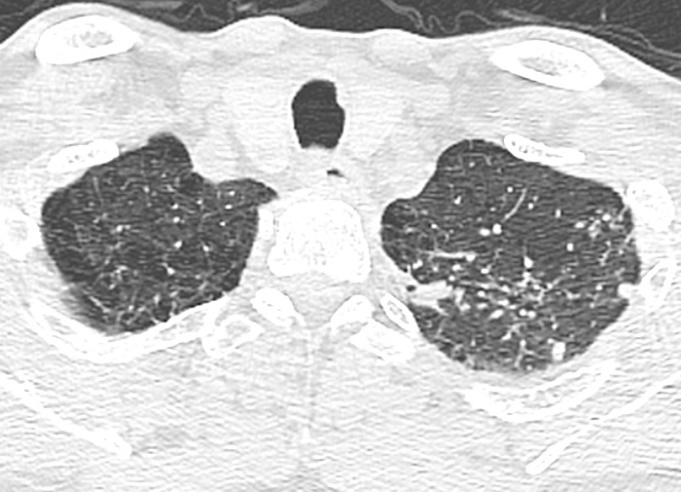  Left upper lobe nodules 2  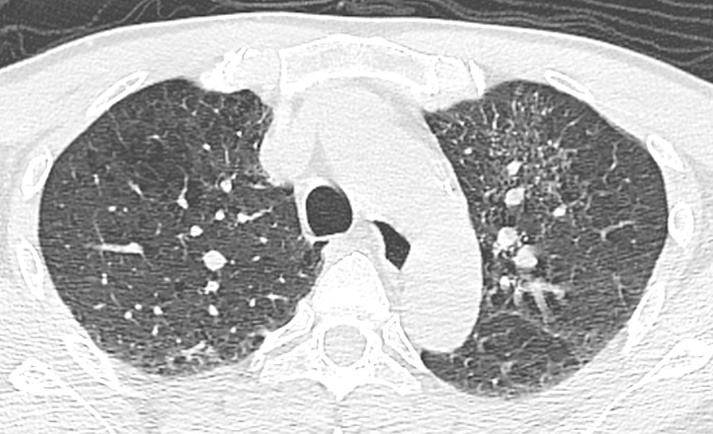  Reticular opacity  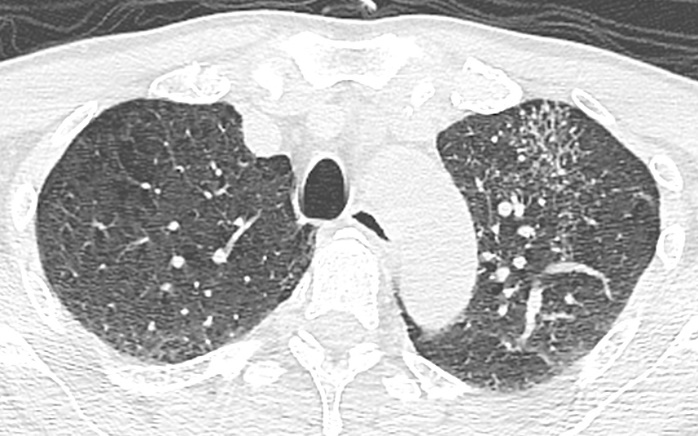  Reticular opacity 2 | 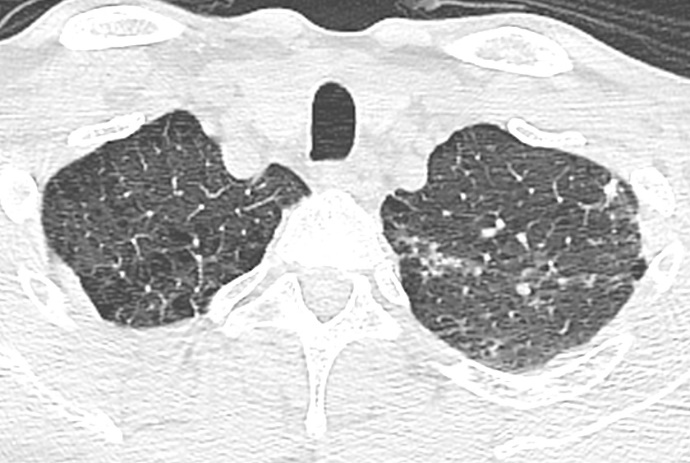  Left upper lobe nodules  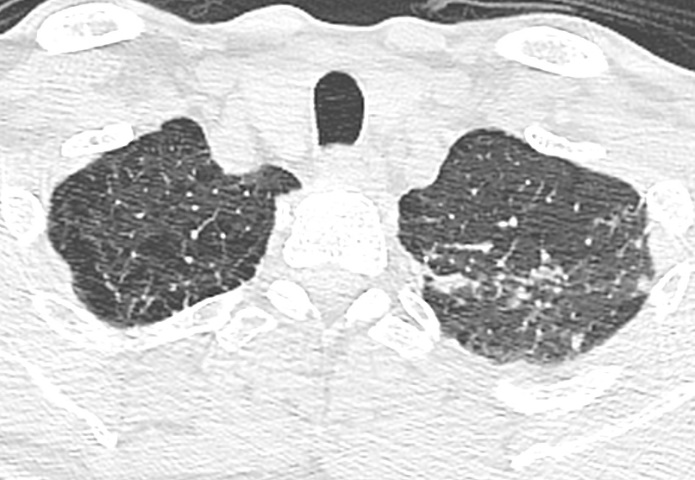  Left upper lobe nodules 2  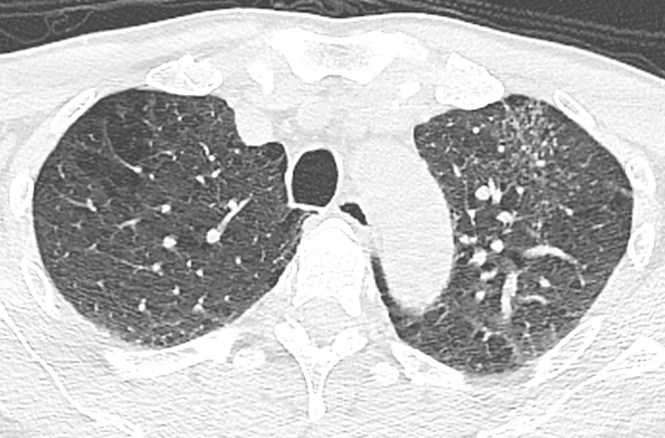  Reticular opacity  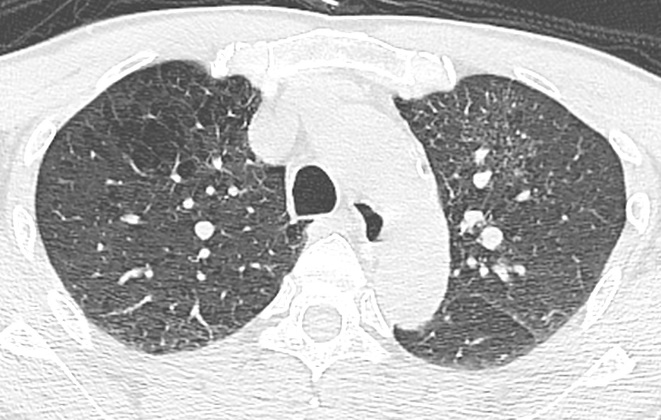  Reticular opacity 2 |

Supplementary Figure 4. Serial PETCT of the lung lesions (a) after 2^nd^ cycle of RVD+Z – June 2021, (b) after 4^th^ cycle of RVD+Z – August 2021, and (c) during lenalidomide maintenance – November 2021

Note:

1. PETCT after 2^nd^ cycle of RVD+Z – July 2021 showed emphysematous lung. There are subpleural and lung nodules in left upper lobe apicoposterior and anterior segment (highest SVmax 4.18, largest 17.9 mm in left apical lung, with evidence of adherence to mediastinal pleura and cavitation). Ground lass lung opacities were seen in left upper lobe anterior segment (SUVmax 1.50) and right lower lobe posterior basal segment (SUVmax 2.9). Left pleural effusion was resolved with mild pleural thickening (SUVmax 1.71).
2. PETCT after 4^th^ cycle of RVD+Z – August 2021 showed stable subpleural and lung nodules in left upper lobe apicoposterior and anterior segment (SUVmax 3.25, largest 13.5 mm in left apical lung, adherence to mediastinal pleura and cavitation remains). Stable ground glass lung opacities in left upper lobe anterior segment (SUVmax 1.93) and right lower lobe posterior basal segment (SUVmax 2.44) and pleural thickening (SUVmax 1.55).
3. PETCT during lenalidomide maintenance – November 2021 showed improved subpleural and lung nodules in left upper lobe apicoposterior and anterior segment (SUVmax 2.17, largest 10.6 mm in left apical lung, adherence to mediastinal pleura and cavitation remains). Improved ground glass lung opacities in left upper lobe anterior segment (SUVmax 1.15) but remain unchanged in extent. Disappearance of previous right lower lobe posterior basal segment lesion. Stable left pleural thickening (SUVmax 1.89).

# Supplementary Figure 5 (Serial MRI Of The Leptomeningeal Myelomatosis)

| (a) | (b) | (c) |
| --- | --- | --- |
| 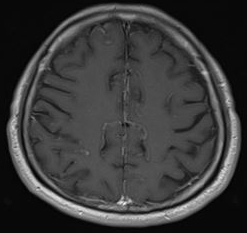  T1 with contrast, vertex level  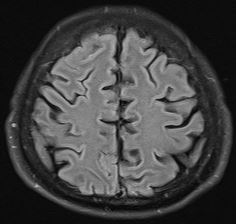  T2 FLAIR, vertex level  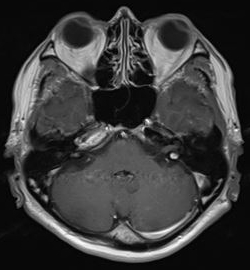  T1 with contrast, level at left IAC lesion  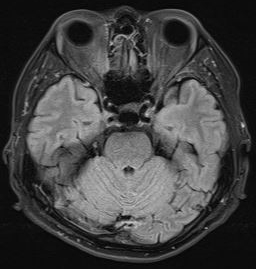  T2 FLAIR, temporal lobe level  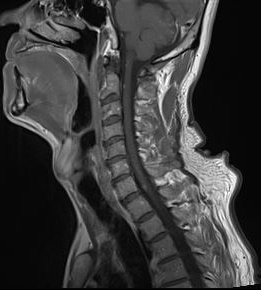  T1 with contrast, cerebellum and cervical spine  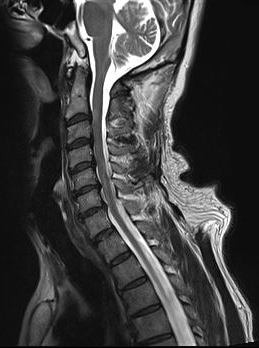  T2, cerebellum and cervical spine  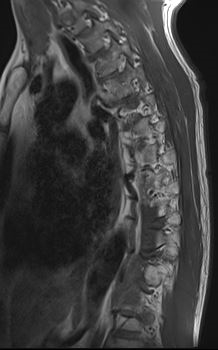  T1 without contrast, thoracic spine  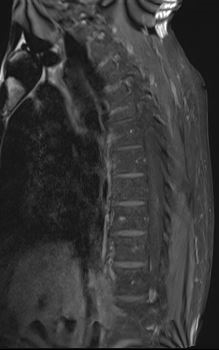  Lesions at T8 level  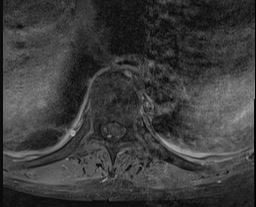  Lesion at T9 level  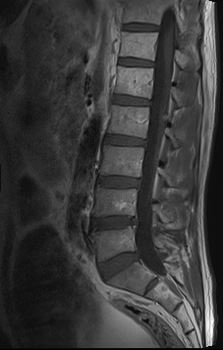  T1 without contrast, lesion at L2 level  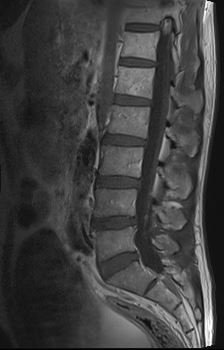  T1 with contrast, lesion at L4 level | 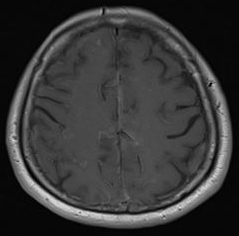  T1 with contrast, vertex level  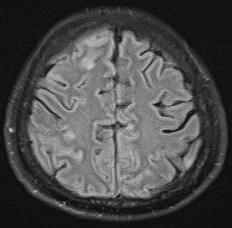  T2 FLAIR, vertex level  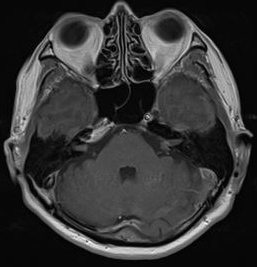  T1 with contrast, level at bilateral IAC lesions  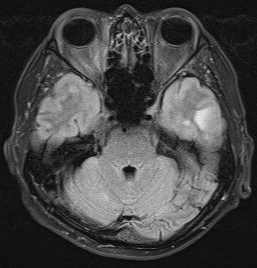  T2 FLAIR, temporal lobe level  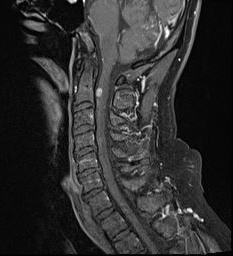  T1 with contrast, cerebellum and cervical spine  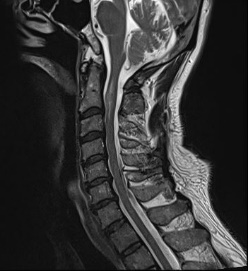  T2, cerebellum and cervical spine | 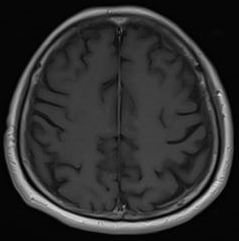  T1 with contrast, vertex level  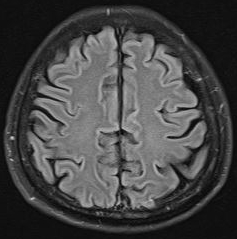  T2 FLAIR, vertex level  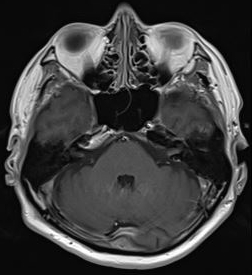  T1 with contrast, level at bilateral IAC lesions  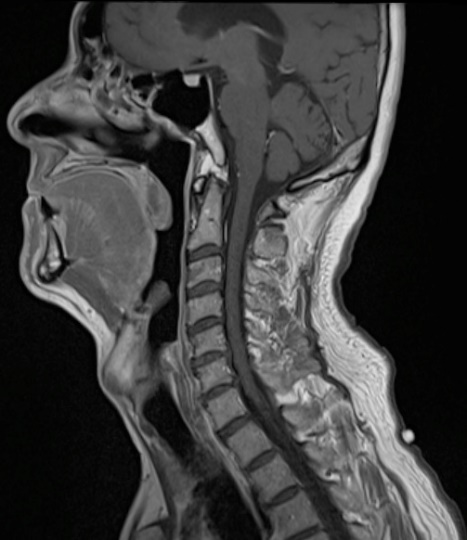  T1 with contrast, cerebellum & cervical spine  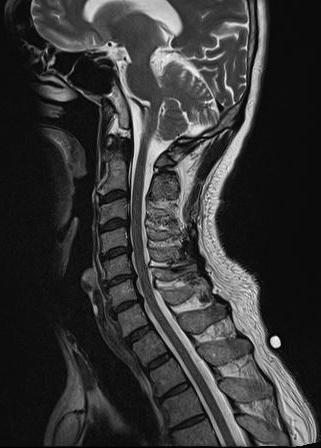  T2, cerebellum and cervical spine  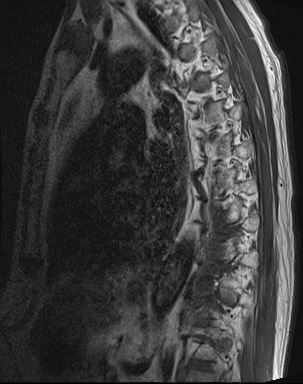  T1 without contrast, thoracic spine  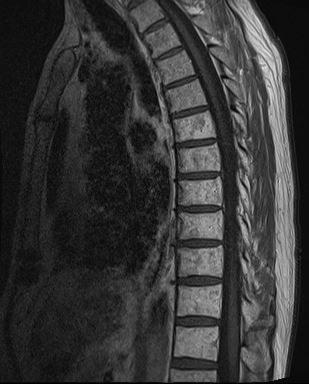  T1 with contrast, lesions at T7 level  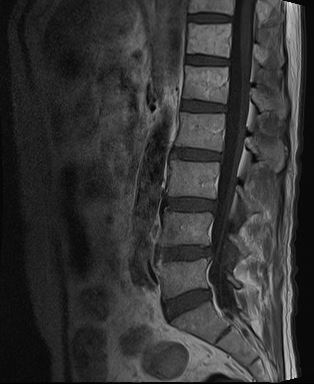  T1 without contrast, lesion at L2 level  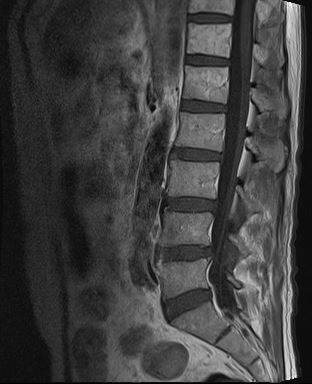  T1 with contrast, lesion at L4 level |

Supplementary Figure 5. Serial MRI of the leptomeningeal myelomatosis (a) at diagnosis of leptomeningeal myelomatosis – February 2022, (b) at progression of leptomeningeal myelomatosis – March 2022, and (c) after 4th cycles of Dara-PVPD – May 2022

Note:

1. MRI at diagnosis of leptomeningeal myelomatosis – February 2022

- Brain
  - Multiple tiny nodular leptomeningeal enhancement scattered over bilateral cerebral hemispheres. They are best seen at right precentral sulcus with three nodular enhancing lesions seen, largest 0.5 x 0.2 cm with high DW1 signal and low ADC signal. The internal auditory canals (IACs) appear normal in configuration. However, there are nodular enhancement within bilateral IACs. It is largest on the left as it occupied entire left IAC, 1.0 x 0.5 cm.
- Whole spine
  - Subtle T2W hyperintensity in anterior cord at C2/C3 and C5-C6 level, likely indicate focal oedema. Focal linear leptomeningeal enhancement noted anterior to the C2 cord oedema area.
  - Diffuse posterior C5/C6 and C6/C7 disc bulge, causing mild cord indentation and left neuroforaminal narrowing. There is mild central canal stenosis (AP diameter 7-8 mm) and possibly left exiting C6 and C7 nerve roots compression.
  - Multiple small nodular leptomeningeal enhancement along the surface of spinal cord. They are noted at C1, C2, lower C7, lower T8, and along cauda equina at L2 and L4 level.
  - Multiple leptomeningeal enhancement noted in bilateral cerebellar region.

1. MRI at progression of leptomeningeal myelomatosis – March 2022

- Brain
  - The leptomeningeal enhancement over bilateral and cerebellar hemispheres is more extensive now.
  - Irregular dural enhancement over bilateral cerebral hemispheres as wee, particularly at right temporooccipital region.
  - Multiple cortical and subcortical oedema scattered over bilateral cerebral hemispheres as well, which were not present in previous MRI. Most of the oedema are adjacent to leptomeningeal nodules.
  - The nodular enhancement within bilateral IACs appears increased in size with the left IAC lesion extending into left cerebellopontine angle, size 1.5 x 0.7 cm.
- Cervical spine
  - Two new oval enhancing lesions over anterior cervical cord at lower C2 level, with cord oedema, size 0.5 x 0.8 x 0.8 cm and the inferior one is 0.3 x 0.4 x 0.6 cm. These are likely arising from previously noted linear leptomeningeal enhancement on MRI February 2022. Adjacent cord oedema from upper C2 till mid C3 level.
  - Several tiny nodular leptomeningeal enhancements along posterior surface of spinal cord at C1 level, and anterior ponto-medullary region. The leptomeningeal enhancement at lower C7 is not well seen in current scan.
  - Diffuse posterior C5/C6 and C6/C7 disc bulge appear similar. It caused mild cord indentation and left neuroforaminal narrowing, with mild central canal stenosis. Left exiting C6 and C7 nerve roots are possibly compressed.
  - Visualised cerebellum also shows the enhancing leptomeningeal nodules has increased in number and size.

1. MRI after 4th cycles of Dara-PVPD – May 2022

- Brain
  - The leptomeningeal and dural meningeal enhancement over bilateral cerebral and cerebellar hemispheres with associated white matter oedema are not seen now.
  - The white matter hyperintense lesion in right external capsule is not well seen now. The lesion in left external capsule is smaller with no enhancement.
  - The nodular enhancement in left IAC appears smaller, size of 0.7 x 0.4 cm. Right IAC lesion is not seen now.
- Whole spine
  - Previously noted leptomeningeal enhancement over cervical, thoracic, and lumbar regions with cord oedema is not seen now.
  - The enhancing marrow lesions in T7-T9 are not well seen now.
  - Diffuse posterior C5/C6 and C6/C7 disc bulge appear similar.

# DeAngelis Protocol

| Drug | Dosage and route | Dosing schedule | |
| --- | --- | --- | --- |
|  |  | **Week** | **Day** |
| Methotrexate | 2,500mg/m^2^ IVI in 500mL D5% over 3H | 1, 3, 5, 7, 9 | D1 |
| Folinic acid rescue | 20mg IV QID with 1^st^ dose at 24H from start of MTX | 1, 3, 5, 7, 9 | D2 – 4 |
| Vincristine | 1.4mg/m^2^ (max 2.8mg) IVI in 100mL NS run fast | 1, 3, 5, 7, 9 | D1 |
| Procarbazine | 100mg/m^2^ PO OD | 1, 5, 9 | D1 – 7 |
| Methotrexate | 12mg Intra-Ommaya or 15mg IT | 2, 4, 6, 8, 10 | D1 |
| Cytarabine | 3,000mg/m^2^ IVI in 500mL NS over 3H | 16, 19 | D1 – 2 |
| Cranial irradiation^1^ | Whole brain 36Gy | 11 |  |
| Dexamethasone | 16mg/day PO in Week 1  12mg/day PO in Week 2  8mg/day PO in Week 3  6mg/day PO in Week 4  4mg/day PO in Week 5  2mg/day PO in Week 6 |  |  |

# R-MPV Protocol

| Drug | Dosage and route | Dosing schedule | |
| --- | --- | --- | --- |
|  |  | **Cycle** | **Day** |
| Rituximab | 500 mg/m^2^ IVI over 5 H | 1 – 5, *6, 7* | D1 |
| Methotrexate | 3,500 mg/m^2^ IVI in 500 ml D5% over 2 H | 1 – 5, *6, 7* | D2 |
| Folinic acid rescue | 20 mg IV QID with 1^st^ dose at 24 H from start of MTX | 1 – 5, *6, 7* | D3 – 5 |
| Vincristine | 1.4 mg/m^2^ (max 2.8 mg) IVI in 100 ml NS run fast | 1 – 5, *6, 7* | D2 |
| Procarbazine | 100 mg/m^2^ PO OD | 1, 3, 5, *7* | D2 – 8 |
| Methotrexate | 12 mg intra-Ommaya or 15mg intrathecal | 1 – 5, *6, 7* | D8 |
| Cytarabine | 3,000 mg/m^2^ IVI in 500 ml NS over 3 H | Week 16, 20 | D1 – 2 |
| Cranial irradiation | See below |  |  |

Cycle interval: 2 weeks.

Patients who obtained a partial response (PR) after 5 cycles received additional 2 cycles of R-MPV.

Intra-Ommaya or intrathecal (IT) methotrexate is administered to patients with positive CSF cytology.

Cranial irradiation:

- Patients who achieve a complete response (CR) after 5 or 7 cycles will receive reduce dose whole brain radiotherapy (rdWBRT) 23.40 Gy (1.8 Gy x 13 fractions) 3 to 5 weeks after chemotherapy completion.
- Opposed lateral radiation fields were used to include the whole brain down to the level of C2 (so-called German helmet shape) and excluded the anterior two thirds of the orbit.
- Patients with PR, stable disease or progressive disease were offered standard WBRT 45 Gy (1.8 Gy in 25 fractions).
- Patients with ocular involvement were irradiated without orbital shielding to the full dose of 23.40 Gy (patients in CR) or to a dose of 36 Gy (patients with less than a CR).

After radiotherapy, all patients received two consolidation high-dose cytarabine cycles (one cytarabine cycle = 28 days).

# Dara-PVPD Protocol

| Drug | Dosage and route | Dosing schedule | |
| --- | --- | --- | --- |
|  |  | **Cycle** | **Day** |
| Daratumumab | 16 mg/kg IVI in 1000 mL NS with infusion rate as per recommendation | 1 – 5, *6, 7* | D1 |
| Pomalidomide | 4mg PO every Monday, Wednesday, Friday | 1 – 5, *6, 7* |  |
| Vincristine | 1.4 mg/m2 (max 2.8 mg) IVI in 100 mL NS over 15 min | 1 – 5, *6, 7* | D1 |
| Procarbazine | 100 mg/m2 PO OD | 1, 3, 5, *7* | D2 – 8 |
| Dexamethasone | 20 mg IV D1 (also as a premedication for daratumumab)  20 mg PO D2  10 mg PO D3  4 mg PO D4  2 mg PO D5 |  |  |
| Thiotepa | 12 mg IT | 1 – 5, *6, 7* | D8 |
| Dexamethasone | 4 mg IT | 1 – 5, *6, 7* | D8 |

Premedication (at least 1 hour prior to daratumumab): paracetamol 1g PO and chlorpheniramine 10 mg IV.

Acyclovir prophylaxis, proton pump inhibitor prophylaxis, and intermittent granulocyte colony-stimulating factor as needed.

Dara-PVPD given in 1^st^ to 6^th^ cycle

|  | Cycle 1 | Cycle 2 | Cycle 3 | Cycle 4 | Cycle 5 | Cycle 6 |
| --- | --- | --- | --- | --- | --- | --- |
| Daratumumab | Per protocol | Per protocol | Per protocol | Per protocol | Per protocol | Per protocol |
| Pomalidomide | Lenalidomide 20 mg every Monday, Wednesday, Friday, Sunday while awaiting arrival of pomalidomide | | Per protocol | Per protocol | Every Wednesday, Thursday and Friday | |
| Vincristine | 2 mg (↓) at D2 | Per protocol | Per protocol | 2 mg (↓) | 2 mg (↓) | 2 mg (↓) |
| Procarbazine | Per protocol | Per protocol | D2 – 5 | D2 – 5 | D2 – 5 | D2 – 5 |
| Dexamethasone | Per protocol | Per protocol | Per protocol | Per protocol | Per protocol | Per protocol |
| Thiotepa | Not given because patient could not be cooperative for the procedure during the first two cycles and was waiting for import permit and arrival of thiotepa. | | | | | |
| Dexamethsone |  |  |  |  |  |  |

Text – modification done
